# 8-Nitro­quinoline

**DOI:** 10.1107/S1600536811010014

**Published:** 2011-03-23

**Authors:** Liang Xu, Bao-Li Xu, Shu-Jun Lu, Bing Wang, Ting-Guo Kang

**Affiliations:** a Liaoning University of Traditional Chinese Medicine, Dalian 116600, People’s Republic of China

## Abstract

The molecule of the title compound, C_9_H_6_N_2_O_2_, is almost planar, with a dihedral angle of 3.0 (9)° between the pyridine and benzene rings.

## Related literature

For the first synthesis of 8-nitro­quinoline, see: Königs (1879 ▶). The crystal studied was synthesised according to the method of Yale & Bernstein (1948 ▶). For the pharmacological activity of quinoline derivatives, see: Franck *et al.* (2004 ▶); Zouhiri *et al.* (2005 ▶). For standard bond lengths, see: Allen *et al.* (1987 ▶).
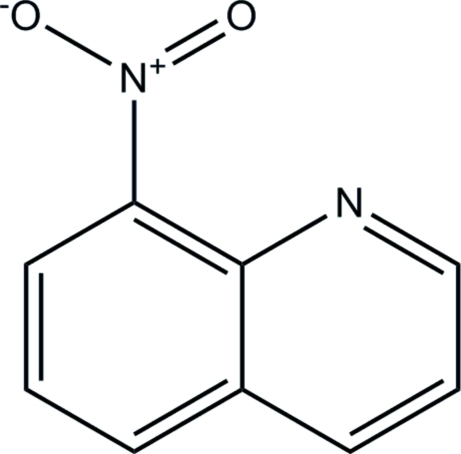


## Experimental

### 

#### Crystal data

C_9_H_6_N_2_O_2_
*M* *_r_* = 174.16
Monoclinic, 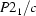

*a* = 7.2421 (11) Å
*b* = 16.688 (3) Å
*c* = 7.2089 (11) Å
β = 114.086 (4)°
*V* = 795.4 (2) Å^3^
*Z* = 4
Mo *K*α radiation
μ = 0.11 mm^−1^
*T* = 296 K
0.40 × 0.32 × 0.25 mm

#### Data collection

Bruker SMART CCD area-detector diffractometer
10084 measured reflections
2287 independent reflections
1827 reflections with *I* > 2σ(*I*)
*R*_int_ = 0.023

#### Refinement

*R*[*F* ^2^ > 2σ(*F* ^2^)] = 0.045
*wR*(*F* ^2^) = 0.147
*S* = 1.03
2287 reflections
119 parameters
H-atom parameters constrained
Δρ_max_ = 0.27 e Å^−3^
Δρ_min_ = −0.22 e Å^−3^

### 

Data collection: *SMART* (Bruker, 2001 ▶); cell refinement: *SAINT-Plus* (Bruker, 2003 ▶); data reduction: *SAINT-Plus*; program(s) used to solve structure: *SHELXTL* (Sheldrick, 2008 ▶); program(s) used to refine structure: *SHELXTL*; molecular graphics: *SHELXTL*; software used to prepare material for publication: *SHELXTL*.

## Supplementary Material

Crystal structure: contains datablocks I, global. DOI: 10.1107/S1600536811010014/hg5010sup1.cif

Structure factors: contains datablocks I. DOI: 10.1107/S1600536811010014/hg5010Isup2.hkl

Additional supplementary materials:  crystallographic information; 3D view; checkCIF report

## Figures and Tables

**Fig. 1.**
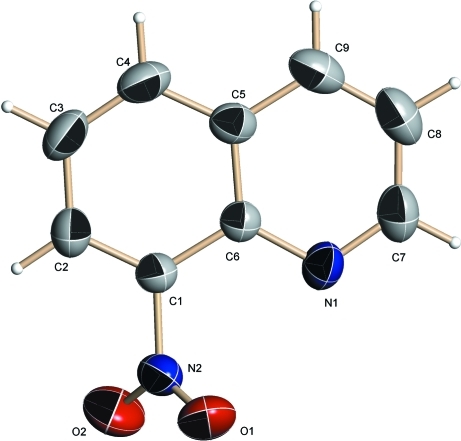
The molecular structure of (I) (thermal ellipsoids are shown at 30% probability levels).

## supplementary crystallographic information

### Comment

8-Nitroquinoline was first synthesized in 1879 (Königs 1879) and in recent years the quinoline and quinoline derivatives have been found to possess a broad spectrum of pharmacological ativity (Franck  *et al.*., 2004, Zouhiri  *et al.*, 2005). However, the crytal structure of 8-Nitroquinoline has not been reported so far. Knowledge of the crystal structure of 8-Nitroquinoline gives us not only information about nuclearity of the complex molecule, but is important in understanding the behaviour of this compounds in the vapour phase, and the mechanisms of sublimation and decomposition. Therefore, we have synthesized the title compound, (I), and report its crystal structure here (Fig. 1).

The bond lengths for (I) are within normal ranges (Allen *et al.*, 1987). The molecule is almost flat, with a dihedral angle of 3.0 (9)° between the pyridine and benzene rings.

### Experimental

The title compound, (I), was prepared according to the literature procedure of Yale & Bernstein (1948). A mixture of 6.96 g(50 mmol) of *o*-Nitrophenol and 14.2 g(100 mmol) of arsenic acid in 50 ml of 86% phosphoric acid was placed in a 250 ml, 3-necked flask fitted with a thermometer, dropping funnel, reflux condenser and magnetic stirrer. The reaction mixture was warmed to 100°C and 4.75 ml(75 mmol) of acrolein added dropwise with vigorous stirring. After all the acrolein had been added, the reaction mixture was stirred for an additional thirty minutes during which time the temperature was maintained at 100°C by warming with an oil bath. The solution was poured into 200 ml of water, treated with Hyflo Supercel and decolorizing carbon and filtered. The filtrate was made alkaline with aqueous ammonia and the precipitated product filtered. The dried solid was refluxed with 150 ml of ethyl acetate and decolorizing carbon,filtered, and concentrated until crystallization started. The product weighed 5.05 g(58% yield). Crystals suitable for X-ray data collection were obtained by recrystallization from dichloromethane–hexane (1:1 *v*/*v*).

### Refinement

H atoms were placed in geometrically idealized positions and allowed to ride on their parent atoms, *C*—H=0.93 for phenyl H atoms, with *U*_iso_(H) = 1.2*U*_eq_(C) for phenyl H.

### Crystal data

**Table:**

C_9_H_6_N_2_O_2_	*F*(000) = 360
*M**_r_* = 174.16	*D*_x_ = 1.454 Mg m^−^^3^
Monoclinic, *P*2_1_/*c*	Mo *K*α radiation, λ = 0.71073 Å
Hall symbol: -P 2ybc	Cell parameters from 3873 reflections
*a* = 7.2421 (11) Å	θ = 3.1–30.0°
*b* = 16.688 (3) Å	µ = 0.11 mm^−^^1^
*c* = 7.2089 (11) Å	*T* = 296 K
β = 114.086 (4)°	Block, yellow
*V* = 795.4 (2) Å^3^	0.40 × 0.32 × 0.25 mm
*Z* = 4	

### Data collection

**Table:**

Bruker SMART CCD area-detector diffractometer	1827 reflections with *I* > 2σ(*I*)
Radiation source: fine-focus sealed tube	*R*_int_ = 0.023
graphite	θ_max_ = 30.0°, θ_min_ = 3.1°
φ and ω scans	*h* = −9→10
10084 measured reflections	*k* = −23→21
2287 independent reflections	*l* = −10→10

### Refinement

**Table:**

Refinement on *F*^2^	Secondary atom site location: difference Fourier map
Least-squares matrix: full	Hydrogen site location: inferred from neighbouring sites
*R*[*F*^2^ > 2σ(*F*^2^)] = 0.045	H-atom parameters constrained
*wR*(*F*^2^) = 0.147	*w* = 1/[σ^2^(*F*_o_^2^) + (0.0809*P*)^2^ + 0.1053*P*] where *P* = (*F*_o_^2^ + 2*F*_c_^2^)/3
*S* = 1.03	(Δ/σ)_max_ < 0.001
2287 reflections	Δρ_max_ = 0.27 e Å^−^^3^
119 parameters	Δρ_min_ = −0.22 e Å^−^^3^
0 restraints	Extinction correction: *SHELXTL* (Sheldrick, 2008), Fc^*^=kFc[1+0.001xFc^2^λ^3^/sin(2θ)]^-1/4^
Primary atom site location: structure-invariant direct methods	Extinction coefficient: 0.133 (14)

### Special details

**Table:**

Geometry. All e.s.d.'s (except the e.s.d. in the dihedral angle between two l.s. planes) are estimated using the full covariance matrix. The cell e.s.d.'s are taken into account individually in the estimation of e.s.d.'s in distances, angles and torsion angles; correlations between e.s.d.'s in cell parameters are only used when they are defined by crystal symmetry. An approximate (isotropic) treatment of cell e.s.d.'s is used for estimating e.s.d.'s involving l.s. planes.
Refinement. Refinement of *F*^2^ against ALL reflections. The weighted *R*-factor *wR* and goodness of fit *S* are based on *F*^2^, conventional *R*-factors *R* are based on *F*, with *F* set to zero for negative *F*^2^. The threshold expression of *F*^2^ > σ(*F*^2^) is used only for calculating *R*-factors(gt) *etc*. and is not relevant to the choice of reflections for refinement. *R*-factors based on *F*^2^ are statistically about twice as large as those based on *F*, and *R*- factors based on ALL data will be even larger.

### Fractional atomic coordinates and isotropic or equivalent isotropic displacement parameters (Å^2^)

**Table:**

	*x*	*y*	*z*	*U*_iso_*/*U*_eq_	
C6	0.25070 (14)	0.69710 (6)	0.26068 (13)	0.0354 (2)	
C1	0.25541 (15)	0.61239 (7)	0.26308 (15)	0.0380 (2)	
N1	0.41946 (14)	0.73928 (6)	0.28382 (15)	0.0454 (3)	
C5	0.06270 (16)	0.73360 (7)	0.22521 (16)	0.0422 (3)	
N2	0.44722 (15)	0.57111 (6)	0.30592 (16)	0.0467 (3)	
C4	−0.10800 (18)	0.68516 (9)	0.1935 (2)	0.0554 (3)	
H4	−0.2304	0.7092	0.1738	0.066*	
C2	0.08895 (19)	0.56627 (8)	0.2262 (2)	0.0502 (3)	
H2	0.0973	0.5107	0.2241	0.060*	
C9	0.0561 (2)	0.81833 (8)	0.21989 (19)	0.0560 (3)	
H9	−0.0634	0.8452	0.1985	0.067*	
C7	0.4030 (2)	0.81781 (8)	0.27567 (19)	0.0547 (3)	
H7	0.5169	0.8474	0.2905	0.066*	
C3	−0.09546 (19)	0.60401 (9)	0.1914 (2)	0.0595 (4)	
H3	−0.2101	0.5732	0.1668	0.071*	
O1	0.59089 (14)	0.58621 (7)	0.46328 (17)	0.0659 (3)	
C8	0.2259 (2)	0.86002 (8)	0.2462 (2)	0.0600 (4)	
H8	0.2248	0.9157	0.2448	0.072*	
O2	0.45132 (17)	0.52226 (7)	0.18178 (19)	0.0742 (4)	

### Atomic displacement parameters (Å^2^)

**Table:**

	*U*^11^	*U*^22^	*U*^33^	*U*^12^	*U*^13^	*U*^23^
C6	0.0357 (5)	0.0374 (5)	0.0308 (4)	−0.0003 (3)	0.0114 (4)	0.0008 (3)
C1	0.0358 (5)	0.0380 (5)	0.0407 (5)	0.0011 (3)	0.0162 (4)	0.0008 (4)
N1	0.0429 (5)	0.0451 (5)	0.0466 (5)	−0.0068 (4)	0.0167 (4)	0.0050 (4)
C5	0.0399 (5)	0.0452 (6)	0.0362 (5)	0.0056 (4)	0.0101 (4)	−0.0023 (4)
N2	0.0459 (5)	0.0398 (5)	0.0594 (6)	0.0052 (4)	0.0266 (4)	0.0074 (4)
C4	0.0334 (5)	0.0679 (8)	0.0594 (7)	0.0041 (5)	0.0132 (5)	−0.0067 (6)
C2	0.0494 (6)	0.0408 (6)	0.0602 (7)	−0.0091 (4)	0.0222 (5)	−0.0052 (5)
C9	0.0630 (8)	0.0481 (7)	0.0494 (6)	0.0180 (5)	0.0154 (5)	−0.0001 (5)
C7	0.0633 (8)	0.0454 (7)	0.0502 (6)	−0.0134 (5)	0.0180 (6)	0.0035 (5)
C3	0.0374 (6)	0.0663 (8)	0.0721 (8)	−0.0153 (5)	0.0196 (6)	−0.0101 (6)
O1	0.0438 (5)	0.0715 (7)	0.0714 (7)	0.0104 (4)	0.0123 (5)	0.0066 (5)
C8	0.0829 (10)	0.0361 (6)	0.0523 (7)	0.0008 (6)	0.0187 (7)	0.0013 (4)
O2	0.0783 (7)	0.0651 (7)	0.0898 (8)	0.0166 (5)	0.0451 (6)	−0.0117 (5)

### Geometric parameters (Å, °)

**Table:**

C6—N1	1.3604 (14)	C4—C3	1.358 (2)
C6—C1	1.4140 (15)	C4—H4	0.9300
C6—C5	1.4163 (14)	C2—C3	1.4035 (19)
C1—C2	1.3619 (16)	C2—H2	0.9300
C1—N2	1.4661 (14)	C9—C8	1.357 (2)
N1—C7	1.3151 (17)	C9—H9	0.9300
C5—C9	1.4148 (18)	C7—C8	1.401 (2)
C5—C4	1.4154 (18)	C7—H7	0.9300
N2—O1	1.2122 (14)	C3—H3	0.9300
N2—O2	1.2198 (15)	C8—H8	0.9300
			
N1—C6—C1	119.99 (9)	C1—C2—C3	118.90 (12)
N1—C6—C5	123.30 (10)	C1—C2—H2	120.6
C1—C6—C5	116.66 (9)	C3—C2—H2	120.5
C2—C1—C6	123.20 (10)	C8—C9—C5	119.35 (12)
C2—C1—N2	117.57 (10)	C8—C9—H9	120.3
C6—C1—N2	119.23 (9)	C5—C9—H9	120.3
C7—N1—C6	116.71 (10)	N1—C7—C8	124.64 (12)
C9—C5—C4	123.30 (11)	N1—C7—H7	117.7
C9—C5—C6	116.99 (11)	C8—C7—H7	117.7
C4—C5—C6	119.70 (11)	C4—C3—C2	120.57 (11)
O1—N2—O2	123.84 (11)	C4—C3—H3	119.7
O1—N2—C1	118.49 (10)	C2—C3—H3	119.7
O2—N2—C1	117.65 (11)	C9—C8—C7	118.98 (12)
C3—C4—C5	120.90 (11)	C9—C8—H8	120.5
C3—C4—H4	119.6	C7—C8—H8	120.5
C5—C4—H4	119.6		
			
N1—C6—C1—C2	−175.08 (10)	C6—C1—N2—O2	−124.71 (12)
C5—C6—C1—C2	2.42 (15)	C9—C5—C4—C3	177.10 (13)
N1—C6—C1—N2	4.57 (14)	C6—C5—C4—C3	−1.69 (19)
C5—C6—C1—N2	−177.94 (9)	C6—C1—C2—C3	−2.46 (18)
C1—C6—N1—C7	178.58 (9)	N2—C1—C2—C3	177.89 (11)
C5—C6—N1—C7	1.26 (15)	C4—C5—C9—C8	−178.13 (12)
N1—C6—C5—C9	−1.77 (15)	C6—C5—C9—C8	0.68 (17)
C1—C6—C5—C9	−179.18 (9)	C6—N1—C7—C8	0.35 (18)
N1—C6—C5—C4	177.09 (10)	C5—C4—C3—C2	1.7 (2)
C1—C6—C5—C4	−0.32 (15)	C1—C2—C3—C4	0.3 (2)
C2—C1—N2—O1	−123.73 (13)	C5—C9—C8—C7	0.76 (19)
C6—C1—N2—O1	56.60 (14)	N1—C7—C8—C9	−1.4 (2)
C2—C1—N2—O2	54.95 (15)		
